# Autophagy as a Regulatory Component of Erythropoiesis

**DOI:** 10.3390/ijms16024083

**Published:** 2015-02-13

**Authors:** Jieying Zhang, Kunlu Wu, Xiaojuan Xiao, Jiling Liao, Qikang Hu, Huiyong Chen, Jing Liu, Xiuli An

**Affiliations:** 1 State Key Laboratory of Medical Genetics & School of Life Sciences, Central South University, Changsha 410078, China; E-Mails: jzhang@nybloodcenter.org (J.Z.); wukunlu@163.com (K.W.); xiaojuan_xiao0809@163.com (X.X.); liaojilingcsu@163.com (J.L.); huqikang1001@163.com (Q.H.); chenhy5000@126.com (H.C.); 2 Red Cell Physiology Laboratory, New York Blood Center, New York, NY 10065, USA; 3 Laboratory of Membrane Biology, New York Blood Center, New York, NY 10065, USA; E-Mail: xan@nybloodcenter.org; 4 College of Life Science, Zhengzhou University, Zhengzhou 450001, China

**Keywords:** autophagy, modulators, erythropoiesis

## Abstract

Autophagy is a process that leads to the degradation of unnecessary or dysfunctional cellular components and long-lived protein aggregates. Erythropoiesis is a branch of hematopoietic differentiation by which mature red blood cells (RBCs) are generated from multi-potential hematopoietic stem cells (HSCs). Autophagy plays a critical role in the elimination of mitochondria, ribosomes and other organelles during erythroid terminal differentiation. Here, the modulators of autophagy that regulate erythroid differentiation were summarized, including autophagy-related (Atg) genes, the B-cell lymphoma 2 (Bcl-2) family member Bcl-2/adenovirus E1B 19 kDa interacting protein 3-like (Nix/Binp3L), transcription factors globin transcription factor 1 (GATA1) and forkhead box O3 (FoxO3), intermediary factor KRAB-associated protein1 (KAP1), and other modulators, such as focal adhesion kinase family-interacting protein of 200-kDa (FIP200), Ca^2+^ and 15-lipoxygenase. Understanding the modulators of autophagy in erythropoiesis will benefit the autophagy research field and facilitate the prevention and treatment of autophagy-related red blood cell disorders.

## 1. Introduction

Erythropoiesis is a continuous and dynamic process by which erythrocytes are generated from multipotent hematopoietic stem cells (HSCs). Erythropoiesis is mainly divided into two stages, early erythroid progenitor proliferation and terminal erythroid differentiation. HSCs proliferate and differentiate into the earliest erythroid progenitors: burst-forming-unit erythroid (BFU-E) cells, and then, colony-forming-unit erythroid (CFU-E) cells. Subsequently, terminal erythroid differentiation starts with proerythroblasts, which undergo three mitoses to produce basophilic, polychromatic, and orthochromatic erythroblasts. Eventually, orthochromatic erythroblasts expel their nucleus and become reticulocytes, which subsequently become mature erythrocytes [1]. Noticeable changes in cellular composition and structure occur during terminal erythroid differentiation, including the filling of the cells with abundant hemoglobin and the clearance of all intracellular organelles from the cells, such as mitochondria and ribosomes [2].

A study performed in 1962 revealed an abundance of membranous structures in murine hepatocytes following treatment with glucagon and found that the mitochondria are degraded by lysosomes [3]. Deter and de Duve first proposed the biological concept of autophagy in an international forum [4]. Autophagy is a key cellular catabolic pathway that can be divided into macroautophagy, microautophagy and chaperone-mediated autophagy, according to the various enveloped substances and transport methods. Macroautophagy is comprised of two types: selective and non-selective. Selective autophagy includes mitophagy and pexophagy, and non-selective autophagy plays an important role in cell starvation [5,6].

Lemasters *et al.* [7] formally proposed the concept of mitophagy in 2005. This group observed that decreasing mitochondrial membrane potentials and the opening of the conductance permeability transition pores of the mitochondrial inner membrane cause mitophagy. The mitochondrion is the powerhouse of the cell, providing almost all energy for cellular activities and generating reactive oxygen species (ROS). ROS may cause damage to mitochondria, releasing apoptosis-inducing factors and leading to cell death. As the major site of biosynthesis (hemoglobin and lipid), the mitochondrion participates in the regulation of intracellular calcium. Therefore, the timely sequestration of damaged mitochondria is important for the normal growth of cells and the maintenance of a stable cellular environment [8,9].

Mitochondrial clearance from reticulocytes occurs through a special process that is regulated by multi-domain autophagy-related protein. The programmed removal of the mitochondria that occurs in reticulocytes represents a physiological model for studying the molecular mechanisms involved in mitophagy [10]. A hemin-induced human myeloid leukemia cell line (K562) has been shown to possess the capacity for erythroid differentiation *in vitro*. Multi-vesicular bodies and autophagy have been observed during K562 cell erythroid maturation [11]. In this review, we summarize the relevant modulators of autophagy involved in the regulation of erythroid differentiation under physiological and pathological conditions.

## 2. Autophagy Regulators and Erythroid Maturation

The targeted deletion of genes related to autophagy has been shown to cause anemia, indicating the presence of defective erythrocyte maturation and impaired mitophagy during terminal erythroid differentiation. The reported autophagy-related regulators that act during erythropoiesis have been summarized in Table 1.

**Table 1:** Autophagy-related modulators in erythropoiesis.

Modulators	Interactions with Other Molecules or Targets	Functions	References
Atg1/Ulk1	Atg13, Hsp90-Cdc37	Regulation of mitochondrial and ribosomal clearance	[12,13,14]
Atg4	-	Fusion of autophagosomes with lysosomes	[15]
Atg7	Atg5	Regulation of mitochondrial removal	[16,17,18,19,20,21]
Nix/Bnip3L	LC3, Atg8, miRNA	Modulation of mitochondrial clearance and autophagosome formation	[22,23,24,25,26,27]
GATA1	FoxO3, LC3-I	Direct regulation of autophagy genes	[28,29,30,31,32,33]
KRAB/KAP1-miRNA	Nix/Bnip3L, Ulk1	Participation in cascade controlling mitophagy	[34]
FIP200	Ulk1, Atg13	Essential autophagy gene in hematopoietic cells	[35,36]
Ca^2+^ and 15-lipoxygenase	-	Ca^2+^ promotes binding of 15-lipoxygenase to modulate the clearance of mitochondria	[37,38,39]

### 2.1. Autophagy-Related Gene (Atg) Family

Many autophagy-related genes have been identified that are critical for selective and/or nonselective autophagic regulatory mechanisms [40]. Atg1 (Ulk1), Atg13 and Atg17 are serine-threonine kinase complexes that regulate the cell cycle and cell growth and proliferation; E1-like enzyme Atg7 can activate Atg12 and conjugate Atg5 and E2-like protein Atg10 to form the preautophagosomal structure. Atg7 also mediates the conjugation of Atg12 to Atg5 and of Atg8 to phosphatidylethanolamine (PE), which participates in the extension of autophagy vesicles [41,42]. Among these genes, Ulk1, Atg4 and Atg7 are reported to play important roles during erythropoiesis.

### 2.2. Uncoordinated 51-Like Autophagy Activating Kinase 1 (Ulk1)

Ulk1, which is a homolog of yeast Atg1, is critical for mitochondrial and ribosomal clearance during erythroid terminal differentiation. The number of reticulocytes, mean cell volume (MCV), mean corpuscular hemoglobin level (MCH), and relative distribution width (RDW) of mature erythroid cells are increased in Ulk1^−/−^ mice. Ulk1^−/−^ reticulocytes exhibit the delayed removal of mitochondria, ribosomes and other organelles *in vitro*, and this defect is overcome via treatment with carbonyl cyanide 3-chlorophenylhydrazone (CCCP), which is a mitochondrial uncoupler that produces ROS and causes membrane depolarization [12,13]. Ulk1 interacts with the Hsp90-Cdc37 complex to promote its stability and activation. In addition, this interaction is conducive to Ulk1-directed phosphorylation and the recruitment of Atg13 to damaged mitochondria. As a Hsp90 antagonist, 17-allylamino-17-demethoxygeldanamycin (17AAG) is the synthetic derivative of geldanamycin, that can inhibit ATP binding and hydrolysis, and block the formation of chaperone complexes. When differentiating erythroid cells are treated with 2.5 μM 17AAG, they display significantly decreased Ulk1 protein levels, but Ulk1 mRNA levels are not affected. Although this treatment does not affect reticulocyte maturation, it notably reduces reticulocytes harboring mitochondria containing autophagosomes. Hsp90-Cdc37, Ulk1 and Atg13 are all required for mitophagy during erythroid differentiation [14].

### 2.3. Autophagy-Related 4 (Atg4)

Autophagy is induced in polychromatic erythroblasts, and autophagosomes remain abundant until enucleation, which stimulates the expression of Atg4 family members (Atg4A and Atg4D) and Atg8. The quantitative electron microscope assay has shown that compared to wild-type, fewer autophagosomes are assembled in Atg4 cysteine mutant Atg4B (C74A)-expressing progenitor cells, suggesting that the roles of Atg4 family members (particularly Atg4B) are important for autophagosome fusion during the differentiation of human erythroblasts [15].

### 2.4. Autophagy-Related 7 (Atg7)

Atg7 plays a critical role in mitochondrial autophagy in the mammalian hematopoietic system and has a unique pro-apoptotic effect on lysosome dysfunction. A previous study has shown that Atg7 is essential for the self-renewal, proliferation and normal functioning of HSCs [16]. According to this study, Vav-Atg7^−/−^ mice showed reductions in hematopoietic stem cells and progenitors of multiple lineages. Furthermore, Atg7-deficient Lin^−^Sca1^+^c-Kit^+^ (LSK) cells accumulate mitochondria and ROS, causing DNA damage, which suggests that mitophagy is important to the regulation of HSCs maintenance. In Atg7^−/−^ erythroid cells, the mitochondria are targeted to form autophagosomes, but autophagosome elongation is impaired, and mitochondrion engulfment is inhibited [16,17]. It has been shown that half of Atg7^−/−^ fetal liver cell-transplanted mice die, and the surviving mice display anemia, reticulocytosis, and lymphopenia [17]. Vav-Atg7^−/−^ mice generated using Atg7 Flox/Flox and Vav-iCre mice have been reported to show severe anemia and shortened lifespans [18]. Additionally, the transferrin receptor is up-regulated, and mitochondrial loss is initiated in Ter119^+^/CD71^−^ elevated cells in the bone marrow of Vav-Atg7^−/−^ mice. The loss of Atg7-mediated mitophagy in Atg7^−/−^ erythroblasts leads to the accumulation of damaged mitochondria with the increased formation of isolation membranes, resulting in cell death. In the absence of Atg7, mitochondrial proteins are selectively removed by mitophagy, but proteins associated with the endoplasmic reticulum and ribosomes are unaffected [18,19]. An additional study has shown that the number of mitochondria and mitochondrial ROS in developing red blood cells (RBCs) are increased in Vav-Atg7^−/−^ mice, and the developing RBCs display phosphatidylserine at their surfaces and undergo caspase 3-mediated apoptosis [20]. A recent study showed that when Atg7 was deleted from erythroid progenitors of wild-type and mtDNA-mutator mice, the genetic disruption of autophagy did not cause anemia in wild-type mice but accelerated the mitochondrial respiration decline and induced macrocytic anemia in the mtDNA-mutator mice [21].

### 2.5. Bcl-2 Family: Bcl-2/Adenovirus E1B 19 kDa Interacting Protein 3-Like (Nix/Binp3L)

The Bcl-2 family is known to play a key role in apoptosis, and its function as a regulator of autophagy has also received increasing interest. Bcl-2 functions as an antiautophagy protein via interacting with the conserved autophagy protein Beclin 1 [43]. Nix (also named Bnip3L), which is a mitochondrial outer membrane protein, is the BH3-only member of the Bcl-2 family that inhibits the proliferation of tumor cell lines [44,45]. Nix activity is mediated through the minimal essential region (MER) in its cytoplasmic domain. The mutation of the central leucine residue of MER causes loss of Nix activity and deters rescue of mitochondrial clearance in reticulocytes [46]. Nix is also a selective receptor that combines with mammalian Atg8 homologs, including microtubule-associated protein light chain 3 (LC3/GABARAP) and ubiquitin-like modifiers, which are indispensable for the maturation of phagophores and autophagosomes [22]. Through its *N*-terminal LC3-interacting region, Nix can recruit GABARAP-L1 to depolarize impaired mitochondria. LC3, which is a mammalian homolog of Atg8, has unmodified (LC3-I) and lipid-modified (LC3-II) forms [23].

In erythroid cells, Nix is upregulated during reticulocyte maturation [47], which is essential for mitochondrial membrane potential dispersion and autophagosome formation. Nix promotes the conversion of LC3-I to LC3-II. In addition, the elimination of the Nix: LC3/GABARAP interaction delays mitochondrial clearance in erythrocytes [24,25]. A previous study has shown that the reticulocytes of Nix^−/−^ mice exhibit markedly abnormal mitochondrial residues. Nix^−/−^ mice display hemolytic anemia and erythroid hyperplasia and increased levels of caspase activation and phosphatidylserine due to the increased production of ROS [25,26]. Nix may function through the ancillary release of cytochrome c or the interaction with other mitochondrial effector molecules. In the absence of Nix, mitochondria are not incorporated into autophagosomes in a timely manner for clearance, leading to an erythroid maturation defect. The Nix-dependent clearance of mitochondria has also been detected in human K562 cells that have been induced to undergo erythroid lineage maturation [26]. Nix may be activated to signal into mitochondria to dissipate their mitochondrial transmembrane potential (ΔΨm) during erythroid cells maturation. Nix interacts with other molecules in mitochondria leading to selective sequestration of mitochondria into autophagosomes [27].

### 2.6. Transcription Factors and KAP1

Transcription factors are regarded as additional essential elements that are in autophagy during erythropoiesis and include erythroid-specific genes, such as GATA1, which play a critical role in erythroid differentiation [28,29]. It has been found that GATA1 directly upregulates the transcription of genes encoding autophagy-related components, such as LC3B and its homologs. In murine erythroid cells, GATA1 activates autophagy-related genes, increasing their expression levels during human erythropoiesis [30]. The forkhead protein FoxO3 is required for the GATA1-mediated induction of LC3 and the formation of autophagosomes in erythroid cells [31,32]. Recently, McIver *et al.* found that GATA-1/FoxO3 could repress the expression of Exosc8, a pivotal component of the exosome complex. When downregulated in primary erythroid precursor cells, Exosc8 could induce erythroid cell maturation [33]. GATA1 establishes a dependent pathway to activate the formation of LC3 and autophagosomes for mitochondrial clearance during erythropoiesis.

KRAB-associated protein 1 (KAP1), which is also named tripartite motif containing 28 (TRIM28), transcription intermediary factor 1 β (TIF1β) or KRAB-interacting protein 1 (KRIP-1), is a transcriptional intermediary factor that acts as a scaffold in transcription complexes. The KRAB/KAP1-miRNA regulatory cascade controls mitophagy during human erythropoiesis [34]. KAP1-depleted erythroblasts exhibit erythrocyte maturation defects and accumulate mitochondria. A luciferase reporter assay performed using mouse erythroleukemia (MEL) cells has shown that miR-351 targets the Nix 3'-UTR. Overexpression of miR-351 inhibits erythroid differentiation and causes mitochondrial accumulation in MEL cells [34]. In human erythroleukemia (HEL) cells, knockdown of Kap1 also leads to the impairment of erythroid differentiation, increased mitochondria and the blockage of autophagy effectors, including Nix. Additionally, hsa-miR-125a-5p expression was increased in KAP1-depleted HEL cells. When hsa-miR-125a-5p is over-expressed, the downregulation of Nix and increased numbers of mitochondria are also observed [34]. Therefore, multi-factorial molecules interact with miRNAs to form a regulatory network during mitophagy for the control of erythropoiesis.

### 2.7. Other Modulators: FIP200, Ca2^+^ and 15-Lipoxygenase

FIP200 (200-kDa focal adhesion kinase family-interacting protein) is known to play an essential role in mammalian autophagy and diverse cellular functions. Several studies have shown that FIP200 is an important part of the Ulk1-Atg13-FIP200 complex in autophagosome formation [35]. The deletion of FIP200 results in increased HSCs cycling, the loss of HSCs reconstituting capacities, aberrant myeloid expansion and the blocking of erythroid maturation. Furthermore, FIP200-null HSCs exhibit abnormal accumulation of mitochondria and have increased ROS levels. These studies suggest that FIP200 is a key regulator of fetal HSCs and plays a potential role in autophagy for the maintenance of fetal hematopoiesis [35,36].

Lipoxygenase is the key enzyme involved in unsaturated fatty acid metabolism, and it can translate arachidonic acid, linoleic acid and other fatty acids into their bioactive metabolites, affecting cell structure, metabolism and signal transduction. 15-lipoxygenase acts in response to oxidative damage and modulates the clearance of mitochondria during reticulocyte maturation [37]. A previous study has indicated that 15-lipoxygenase sediments with the mitochondrial fraction in rabbit reticulocytes [38]. Ca^2+^ promotes the binding of 15-lipoxygenase to reticulocyte mitochondria and stimulates the lipid peroxidation of mitochondrial lipids and free linoleic acid. Therefore, Ca^2+^ is important for regulating the 15-lipoxygenase-mediated degradation of mitochondria in reticulocytes [39].

**Figure 1:**
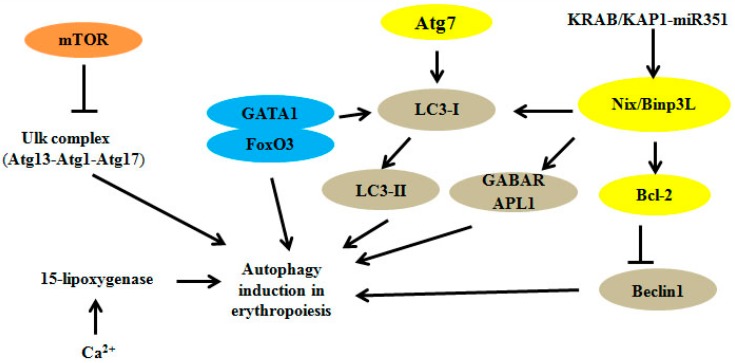
Autophagy-related factors are involved in the regulation of signal pathways in erythroid cells. The mTOR pathway is an important pathway that directly modulates the Ulk1 complex, and the inhibition of mTOR represses autophagy-related processes. Atg7 and Nix/Bnip3L are required for the removal of mitochondria, inducing the conversion of LC3-I to its lipid‑modified form, LC3-II, to promote autophagy. miRNAs can regulate the expressions of key transcriptional components, and Ca^2+^ promotes the binding of 15-lipoxygenase to reticulocyte mitochondria.

Mammalian autophagy signaling pathways are complicated and the mTOR-dependent pathway is the most prominent. AMPK (AMP-activated protein kinase) and mTOR (mammalian target of rapamycin) regulate autophagy through the direct phosphorylation of Ulk1. In nutrient-deficient conditions, AMPK promotes autophagy through the activation of Ulk1, and conversely, mTOR activity prevents Ulk1 activation under normal situations [48]. The implicated roles of the intracellular autophagy pathway in erythroid cell differentiation and maturation are summarized in Figure 1.

## 3. Autophagy and β-Thalassemia

Several recent reports have indicated the key role of autophagy in red cell disorders, including β-thalassemia and myelodysplasia syndrome (MDS) [49,50,51]. When cultured CD34^+^ erythroid progenitor cells from peripheral blood obtained from normal and β-thalassemia patients are induced to erythroid differentiation, autophagy is increased in the erythroblasts from the β-thalassemia patients compared with the normal erythroblasts, and this increase is mediated by the high levels of Ca^2+^ in the β-thalassemia erythroblasts. Normal erythroblasts show increased apoptosis following treatment with l-asparagine, which is an autophagy inhibitor, but this is not observed in erythroblasts from patients with β-thalassemia. Furthermore, reduced Ca^2+^ levels cause decreases in both autophagy and apoptosis. The high levels of autophagy may contribute to the increased apoptosis, leading to anemia and ineffective erythropoiesis in erythroblasts from β-thalassemia patients [51]. Notably, it has also been demonstrated that the occurrence of autophagy and early differentiation are linked in hESCs [52].

## 4. Perspectives

Autophagy is important in maintaining a cellular homeostatic environment. Autophagy has been implicated in many diseases, including various cancers, central nervous system (CNS)-related disorders, neurodegenerative disorders and heart disease in addition to aging. Autophagy studies are developing rapidly in the field of biology, especially in the erythroid research field. If autophagy is impaired during erythroid differentiation, erythroid maturation will be deficient. In this review, we summarize the relevant modulators of autophagy involved in the regulation of erythroid differentiation, which imply that autophagy is an important and complex process during erythroid differentiation. Understanding the modulators of autophagy in normal and pathologic erythropoiesis may facilitate the prevention and treatment of red blood cell-related disorders.

## Abbreviation

17AAG: 17-allylamino-17-demethoxygeldanamycin
AMPK: AMP-activated protein kinase
Atg: autophagy-related
Bcl-2: B-cell lymphoma 2
BFU-E: burst-forming-unit erythroid
CCCP: carbonyl cyanide 3-chlorophenylhydrazone
Cdc37: cell division cycle 37
CFU-E: colony-forming-unit erythroid
CNS: central nervous system
FIP200: focal adhesion kinase family-interacting protein of 200-kDa
FoxO3: forkhead box O3
GABARAP: gamma aminobutyric acid A receptor-associated protein
GATA1: globin transcription factor
HEL: human erythroleukemia
HSCs: hematopoietic stem cells
Hsp90: 90 kDa heat shock protein
KRAB: Krueppel-associated box
KAP-1: KRAB-associated protein 1
KRIP-1: KRAB-interacting protein 1
LC3: light chain 3
LSK: Lin^−^Sca1^+^c-Kit^+^
MCV: mean cell volume
MCH: mean corpuscular hemoglobin
MDS: myelodysplasia syndrome
MEL: mouse erythroleukemia
MER: minimal essential region
mTOR: mammalian target of rapamycin
Nix/Binp3L: Bcl-2/adenovirus E1B 19 kDa interacting protein 3-like
PE: phosphatidylethanolamine
RBCs: red blood cells
ROS: reactive oxygen species
RDW: relative distribution width
TRIM28: tripartite motif containing 28
TIF1β: transcription intermediary factor 1 beta
Ulk1: uncoordinated 51-like autophagy activating kinase 1
